# Reinforcement of a Subcutaneous Pocket for Implantable Cardioverter Defibrillator Insertion Using Acellular Dermal Matrix: A Case Report

**DOI:** 10.3390/jcm13092614

**Published:** 2024-04-29

**Authors:** Jun Ho Choi, Ho Jun Lee, Kwang Seog Kim, Hyung Wook Park, Insu Choi, Jae Ha Hwang, Sam Yong Lee

**Affiliations:** 1 Department of Plastic and Reconstructive Surgery, Chonnam National University Hospital, Chonnam National University Medical School, Gwangju 61469, Republic of Korea; 2 Division of Cardiovascular Medicine, Department of Internal Medicine, Chonnam National University Hospital, Chonnam National University Medical School, Gwangju 61469, Republic of Korea; 3 Department of Pediatrics, Chonnam National University Hospital, Chonnam National University Medical School, Gwangju 61469, Republic of Korea

**Keywords:** acellular dermal matrix, child, defibrillators, implantable, reinforcement, subcutaneous pocket

## Abstract

Pediatric patients who undergo implant insertion into the chest wall face a high risk of implant exposure to the external environment. Five months after an 8-year-old boy underwent implantable cardioverter–defibrillator (ICD) implantation in a subcutaneous pocket in the left anterolateral chest wall to manage long QT syndrome, ICD replacement became necessary owing to exposure risk from distal and lateral thinning of the ICD pocket. Pocket rupture and exposure would increase the risk of infection; therefore, we performed ICD removal and primary pocket closure. Two weeks later, a new suprafascial pocket was created, an acellular dermal matrix (ADM) was attached to the inner wall to prevent ICD protrusion, and a new ICD was inserted. One year postoperatively, the ADM was engrafted, and no complications were observed. A thin subcutaneous layer increases the risk of ICD implantation complications. Inner wall strengthening with an ADM can help prevent pocket rupture.

## 1. Introduction

In the management of long QT syndrome (LQTS), β-adrenergic blocking agents, left cardiac sympathetic denervation, and implantable cardioverter–defibrillator (ICD) placement are commonly utilized. The general consensus in cases of documented cardiac arrest is to prompt implantation of an ICD, regardless of any ongoing treatments [1]. The intention of ICD placement in children with inherited arrhythmia syndromes such as LQTS or Brugada syndrome is prolongation of life. ICD implantation, however, is associated with risks, including potential infection, malfunction, pocket complications, hematoma, and pneumothorax [2]. Children have smaller thoracic cavities than adults do, along with thinner soft tissue in the chest wall. Therefore, when children receive large thoracic implants, they are at increased risk of implant exposure. Olde Nordkamp et al. [2] found that 22% of 4916 pediatric patients experienced ICD-related complications, with 1.6% requiring reinterventions because of pocket complications.

Since the report of the initial study on breast reconstruction using an acellular dermal matrix (ADM) as an implant was published in 2001, numerous subsequent reports have suggested that an ADM can help reduce the risk of capsular contracture and maintain the shape of the reconstructed breast [3]. Additionally, a case series reported by Baxter in 2003 indicated that the use of an ADM in breast surgery can augment atrophied tissue and reinforce the capsule [4]. However, to date, the use of ADM in ICD implantation has not been reported.

In this report, we present the case of an 8-year-old boy diagnosed with LQTS. The boy experienced skin erosion over his ICD site. This issue was successfully resolved by reinserting the ICD using an ADM.

## 2. Case Presentation

**Figure 1:**
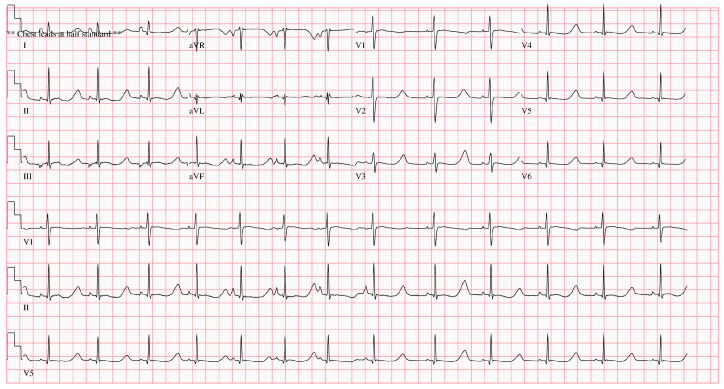
An 8-year-old boy had an implantable cardioverter–defibrillator inserted in a subcutaneous pocket of the left anterolateral chest wall 5 months previously for the treatment of long QT syndrome. Electrocardiogram at the time of the patient’s diagnosis with long QT syndrome.

**Figure 2:**
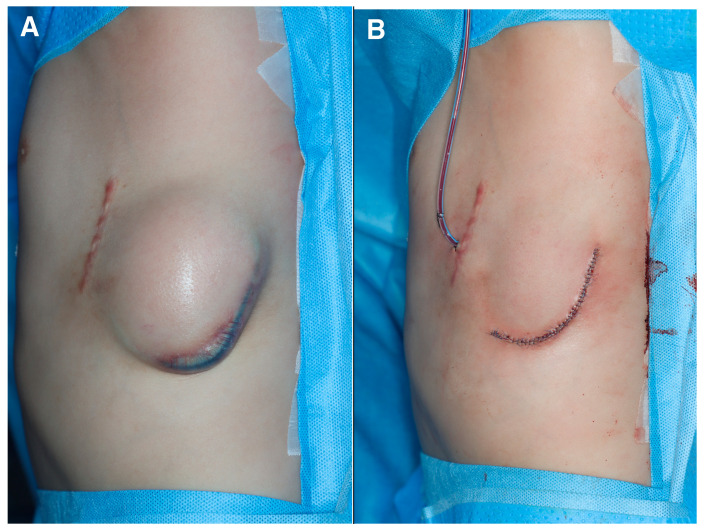
Initial intraoperative photographs. (**A**) Image depicting the impending state in which the implantable cardioverter–defibrillator (ICD) is descending, causing thinning of the chest skin and imminent external exposure. (**B**) Immediate postoperative photograph of ICD removal.

**Figure 3:**
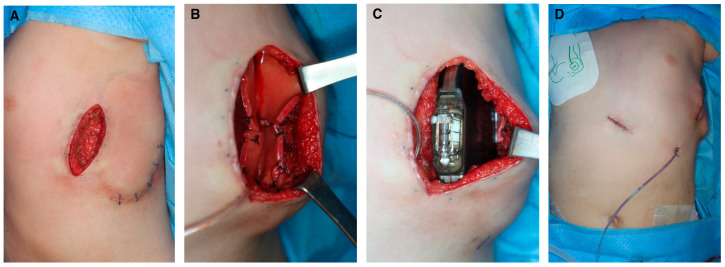
Intraoperative photographs from the second operation. (**A**) Approaching the incision site from previous cardiological interventions to form a suprafascial pocket. (**B**) Acellular dermal matrix attached to the inner wall of the pocket to prevent implantable cardioverter–defibrillator (ICD) protrusion. (**C**) Newly inserted ICD. (**D**) Immediate postoperative photograph of ICD reinsertion.

**Figure 4:**
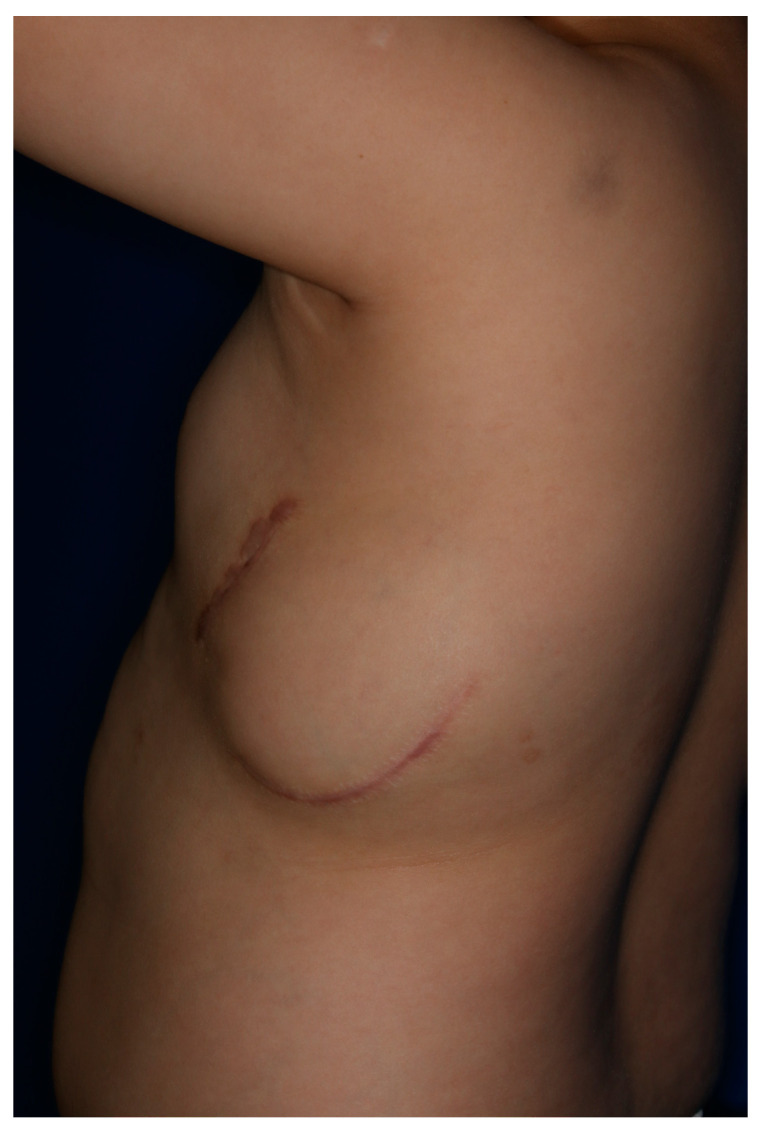
One-year-postoperative photograph.

An 8-year-old boy who underwent cardiopulmonary resuscitation after a cardiac arrest was diagnosed with LQTS (type 8, CACNA1C gene mutation) and subsequently underwent implantation of an ICD for secondary prevention (Figure 1). The ICD was implanted in a subcutaneous pocket of his left anterolateral chest wall via two incisions, with preservation of the muscle fascia, at our institution’s cardiology department. The pocket healed uneventfully and resumed proper function. Five months after implantation, thinning of the distal and lateral aspects of the ICD pocket was observed. The cardiologists consulted the plastic surgery department with the request to replace the ICD because of concerns about device exposure. A rupture of the pocket and subsequent exposure to the external environment would significantly increase the risk of infection; therefore, the ICD was removed and the pocket was primarily closed (Figure 2). The scar from the initial ICD insertion was preserved, while debridement was performed on the area where the skin had thinned due to the descending ICD. This facilitated ICD reinsertion. Two weeks later, with the absence of inflammation confirmed, a new pocket was created in the suprafascial layer at the same location as the original implantation. An ADM was attached to the inner wall of the pocket to prevent ICD protrusion, and a new ICD was inserted (Figure 3). The ICD (EMBLEM A219, Boston Scientific Corp., Marlborough, MA, USA) measured 83.1 × 69.1 × 12.7 mm, weighed 130 g, and had a volume of 59.5 cm^3^. The ADM (AlloDerm RTU, Allergan Inc., Madison, NJ, USA) used for this procedure measured 20 × 10 × 0.24 cm. Postoperatively, it was necessary for the patient to consistently wear specialized supportive clothing to counteract gravitational forces and prevent skin erosion. One year after the operation, the ADM had successfully engrafted, and no complications, such as seroma formation, inflammation, or pocket rupture, were observed (Figure 4).

## 3. Discussion

The recommended pocket position for the pulse generator of a subcutaneous ICD is between the anterior axillary line of the fifth intercostal space and the midaxillary line. The subcutaneous lead is situated parallel to the sternum on the left side and is anchored at the xiphoid process level. The subcutaneous ICD employs a modified surface electrocardiogram, creating three vectors between the two sensing electrodes and the pulse generator to detect ventricular arrhythmias. When a ventricular arrhythmia is detected, the device charges 80 J of energy and delivers a biphasic waveform defibrillating shock [5]. In this case, a pediatric patient who had previously been successfully resuscitated from sudden cardiac arrest was diagnosed with LQTS. In accordance with the prescribed treatment algorithm, a subcutaneous ICD was successfully inserted.

When considering ICD insertion, clinicians can choose between the subcutaneous ICD and the transvenous ICD. Of the two insertion methods, the subcutaneous ICD has demonstrated superior safety and efficacy in young patients, boasting lower complication rates as compared to the transvenous ICD. The latter carries the risk of significant lead-related complications and potential venous access issues, posing significant concerns when used mid-term in young patients [6]. In this case, subcutaneous insertion was prioritized, considering the young age of the pediatric patient. When thinning of the ICD pocket was observed, creation of a new pocket in the upper chest for a smaller-sized pulse generator for the transvenous ICD was primarily considered. However, this option was eventfully withdrawn, considering the potential complications of a transvenous ICD in pediatric patients, in favor of reinforcement and reuse of the existing subcutaneous pocket.

**Figure 5:**
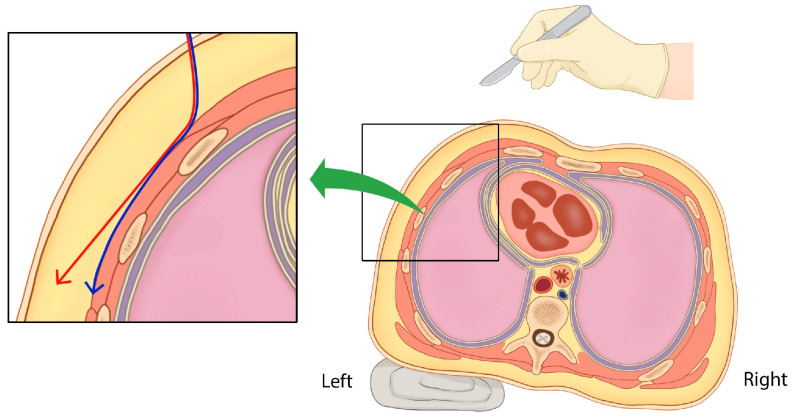
Transverse section of the thorax at cardiac level. Proper (blue arrow) and improper (red arrow) directions of subcutaneous dissection for subcutaneous ICD insertion. To ensure an adequate view inside the ICD pocket on the left lateral chest, it is advisable for a right-handed surgeon standing on the patient’s right side to tilt the patient 30 degrees to the right.

There are several precautions to consider when inserting a subcutaneous ICD in thin people. When making an incision in the anterior chest and performing subcutaneous dissection laterally at the suprafascial level, it is important to note that there is a risk of thinning the pocket’s deepest subcutaneous layer (Figure 5). In our case, when skin erosion occurred after the initial ICD insertion, the subcutaneous pocket was thinnest at its most inferior aspect owing to the influence of gravity. Inadequate dissection also resulted in thinning of the tissue in the superficial portion of the pocket’s deepest area (Figure 2). Children’s thoraxes have smaller anteroposterior-to-transverse diameter ratios and are rounder than adult thoraxes [7]. Therefore, caution is required when performing pocket dissection for ICD insertion, as the angle of dissection gradually steepens laterally. When operating on thin people, especially children, collaborating with plastic surgeons proficient in handling soft tissues could lower the complication rates.

Furthermore, research using ultrasound and X-ray imaging has demonstrated that the thickness of the skin and subcutaneous fat in the chest wall increases with age. Children, therefore, typically have thin layers of skin and fat [8,9]. Patients who lack sufficient subcutaneous tissue coverage for ICD devices may be at a higher risk of experiencing pocket-related complications, such as infection and skin erosion [10]. There are various surgical techniques available to reinforce soft tissue structures, including fat grafts and dermofat grafts. However, given the potential for donor site morbidity among children (who are still growing), using autologous tissues may not be the optimal choice [11]. In our case, when considering the ICD reimplantation, we contemplated dissecting the serratus anterior or latissimus dorsi muscles to transition the subcutaneous pocket to an intramuscular level. However, this was not a viable option for effective chest wall reinforcement because of the thinness of pediatric muscles. Furthermore, selecting an intramuscular pocket was not considered as a solution because continuous pressure from the ICD directly above the bone could lead to deformation of the bone. For thin people, even adults, this may not be an effective option for the same reasons.

In a study of 6433 ICD patients, Ezzat et al. [12] reported incidences of pocket infection in 1.5% of patients, and of hematoma in 1.2%. If there is potential for infection in the existing pocket, reinserting an ICD may increase the risk of complications. Although no studies have yet specifically addressed the rate of seroma occurrence after reinserting an ICD into a pocket with infection potential, Kraenzlin et al. [13] observed that patients who experienced infections accompanied by seroma or necrosis during insertion of the primary tissue expander for breast reconstruction required more frequent hospital visits and surgical procedures during second stage reconstruction. The majority of complications following ICD implantation are related to the pocket. Only one case of skin erosion caused by the inserted defibrillator lead is known [14]. In our case, the possibility of pocket infection due to skin erosion caused by the ICD was considered. To completely eliminate this risk, the absence of infection in the surgical site was confirmed for at least 2 weeks through physical examinations and blood tests after removing the ICD body and the cable. Subsequently, the existing pocket was expanded and safely reinforced with an ADM, and the ICD was successfully reinserted. 

ADMs were developed to improve the properties of the original extracellular matrix and promote organized regeneration of host tissue in various clinical contexts [15]. ADMs and synthetic mesh are frequently used to reinforce soft tissue structures [16]. An ADM is a biological graft material obtained from decellularized human cadaveric tissue or animal dermis. The process of cellular component removal minimizes the risk of eliciting an immune response in the recipient. However, ADMs retain the structural and functional properties of the dermis, including the basement membrane, cellular matrix, and collagen fibers. As a biological scaffold, an ADM encourages angiogenesis and speeds up tissue ingrowth and cellular repopulation, leading to tissue regeneration [17,18]. 

ADMs can be used to provide soft tissue support to optimize breast volume and shape, assist in stabilizing the implant pocket, reinforce the skin flap, and more precisely define the inframammary fold in implant-based breast reconstruction [19]. Moreover, Gill et al. [20] reported the successful utilization of an ADM to reinforce soft tissue, thereby preventing the exposure of vertical expandable prosthetic titanium ribs in pediatric patients. Furthermore, an ADM acts as a blueprint for the formation of neodermis tissue, offering an advantage in postoperatively minimizing scar contracture [21]. Despite the high costs associated with ADMs, particularly considering the limited insurance coverage for its use in conjunction with ICD implantation, this case report is significant because it marks the first documented instance of an ADM being used to reinforce the pocket for ICD insertion. This highlights the potential role of ADMs in addressing skin erosion resulting from the ICD implantation process.

To enhance the successful maintenance of an ICD, several external factors must be considered. First, designing the ICD device to be thinner, with a reduced surface area and lighter weight, would likely decrease the likelihood of skin erosion. Second, crafting the ICD to have an appropriate concave shape (rather than flat) to conform to the chest wall’s curvatures, could reduce the chance of the device’s ends protruding from the pocket and causing skin thinning. Third, when creating a subcutaneous pocket on the left side of a patient’s chest, a task typically performed by a right-handed surgeon from the patient’s right side, tilting the patient approximately 30 degrees to the right during surgery may allow for dissection at the suprafascial level. This positioning helps the surgeon maintain a consistent skin envelope thickness and provides adequate visibility when dissecting areas distant from the pocket opening (Figure 5). These measures can prevent incorrect dissection angles and ensure sufficient visibility for the surgeon when dissecting areas far from the pocket opening.

## 4. Conclusions

When inserting a subcutaneous ICD in pediatric and low-body-mass-index patients, reinforcing the subcutaneous pocket with an ADM may help minimize complications such as skin erosion.

## Data Availability

All data created or analyzed in this study are included in this published article.

## Abbreviations

ADM = acellular dermal matrix, ICD = implantable cardioverter–defibrillator, LQTS = long QT syndrome.
